# Bipolar disorder, Deafness and Culturality in Psychiatric Home Hospitalization: A Clinical Case

**DOI:** 10.1192/j.eurpsy.2024.901

**Published:** 2024-08-27

**Authors:** J. Marti Bonany, G. De Iturbe Catania, R. Romar Navia, D. Tolosa Merlos, C. Montserrat Diez, E. Pechuan Martínez, R. Rodriguez Seoane, L. Gil Martinez, D. Garcia Hernandez

**Affiliations:** Salut Mental Institut, Hospital del Mar, Barcelona, Spain

## Abstract

**Introduction:**

Mental health in the deaf community is a complex issue. Challenges in diagnosis and treatment arise from a lack of experienced interpreters and difficulties in translating Sign Language to spoken language. Deaf individuals, due to auditory limitations, are more vulnerable to abuse, increasing their risk of mental health disorders, including bipolar affective disorder (BPAD). BPAD is a prevalent, debilitating condition with varied prevalence estimates. Managing it is tough due to its lifelong, unpredictable nature. A new approach called Psychiatric Home Hospitalization Unit aims to provide acute mental health care at home as an alternative to hospitalization.

**Objectives:**

To show the management of severe bipolar disorder with comorbidity from a Psychiatric Home Hospitalization Unit

**Methods:**

A clinical case of bipolar disorder with deafness attended at the Psychiatric Home Hospitalization Unit of our hospital is presented.

**Results:**

A 24-year-old deaf woman borned in Pakistan and later moving to Catalonia, she faced educational challenges but ultimately completed her studies with sign language support. Afterward, she struggled to find suitable employment, and her family had a history of bipolar disorder.

She exhibited a sudden change in behavior, characterized by irritability, paranoia, and distrust. Communication was challenging due to her speech difficulties, but assessments using sign language and observation were conducted. Her physical examination was normal, but her speech was disorganized and pressured, suggesting possible auditory hallucinations and thought disturbances. She was hospitalized and diagnosed with bipolar disorder with psychotic features.

During her initial hospitalization, she received lithium, olanzapine, clotiapine and benzodiacepines. After discharge, she continued treatment through a home hospitalization service during almost 4 month. During follow-up she presented a course with high affective instability, rapid cycling alternating brief periods of stability with other presenting manic and mixed features with high disorganization.

Due to the rapid cycling pattern Valproic acid was considered. Valproic acid was introduced up to 700 mg/d (97.1 mcg/mL). Treatment with lithium carbonate 800 mg/d (0.91 mEq/L) was maintained. Previous antipsychotic regimen was changed to quetiapine 400mg/d, olanzapine 5mg/d. Her condition improved significantly with the adjusted treatment regimen. She was discharged to an outpatient service.

**Conclusions:**

Diagnosing and treating bipolar affective disorder (BPAD) in a deaf and mute patient posed unique challenges. The rapid mood cycling pattern and complexity of her case made treatment challenging. Family information and interpreter support were vital. Cultural factors were considered, and home hospitalization was crucial in managing symptoms that lasted over four months.

**Disclosure of Interest:**

None Declared

